# Risk Factors for Acute Leukemia in Children: A Review

**DOI:** 10.1289/ehp.9023

**Published:** 2006-11-30

**Authors:** Martin Belson, Beverely Kingsley, Adrianne Holmes

**Affiliations:** Centers for Disease Control and Prevention, National Center for Environmental Health, Division of Environmental Hazards and Health Effects, Health Studies Branch, Atlanta, Georgia, USA

**Keywords:** acute, ALL, AML, children, environment, leukemia, risk factors

## Abstract

Although overall incidence is rare, leukemia is the most common type of childhood cancer. It accounts for 30% of all cancers diagnosed in children younger than 15 years. Within this population, acute lymphocytic leukemia (ALL) occurs approximately five times more frequently than acute myelogenous leukemia (AML) and accounts for approximately 78% of all childhood leukemia diagnoses. Epidemiologic studies of acute leukemias in children have examined possible risk factors, including genetic, infectious, and environmental, in an attempt to determine etiology. Only one environmental risk factor (ionizing radiation) has been significantly linked to ALL or AML. Most environmental risk factors have been found to be weakly and inconsistently associated with either form of acute childhood leukemia. Our review focuses on the demographics of childhood leukemia and the risk factors that have been associated with the development of childhood ALL or AML. The environmental risk factors discussed include ionizing radiation, non-ionizing radiation, hydrocarbons, pesticides, alcohol use, cigarette smoking, and illicit drug use. Knowledge of these particular risk factors can be used to support measures to reduce potentially harmful exposures and decrease the risk of disease. We also review genetic and infectious risk factors and other variables, including maternal reproductive history and birth characteristics.

Although overall incidence is rare, leukemia is the most common type of childhood cancer, accounting for 30% of all cancers diagnosed in children younger than 15 years (Linet 1999). Within this population, acute lymphocytic leukemia (ALL) occurs approximately five times more frequently than acute myelogenous leukemia (AML) and accounts for approximately three-quarters of all childhood leukemia diagnoses (Gurney et al. 1995; Pui 1997, 2000; Zipf et al. 2000). Chronic myeloid leukemias make up most of the other childhood leukemia cases. Childhood cases account for about 12% of all leukemias but make up > 60% of ALL cases (Sandler and Ross 1997).

In the United States, the incidence of childhood ALL increased between 1975 and 2002; however, this increase was not statistically significant. The observed increase may be due in part to changes in diagnostic practice and accuracy and improved reporting. The incidence rate of childhood ALL is now stable at three to four new cases per year per 100,000 children who are younger than 15 years, with a peak incidence at approximately 2–5 years of age (Greenlee et al. 2000; Gurney et al. 1995; Margolin et al. 2001; Ross et al. 1994; Swensen et al. 1997). For childhood AML, the peak incidence rate occurs during the first year of life then decreases steadily through 4 years of age, where it remains relatively constant throughout childhood (Gurney et al. 1995).

The incidence of ALL, particularly T-cell ALL, is slightly higher among boys than girls. (Kersey et al. 1973; Margolin et al. 2001; Ries et al. 1998; Zahm and Devesa 1995). Girls, however, have a higher incidence of all leukemias during the first year of life (Gurney et al. 1995; Ries et al. 1998). No differences in incidence of AML exist between girls and boys.

Throughout childhood, the incidence rate of ALL among African American children is approximately half the rate among Caucasian children. During the first few years of life, the incidence rate of AML among African American children is approximately one-third the rate among Caucasian children; however, African American children ≥ 3 years of age have higher rates than Caucasians (Gurney et al. 1995; Pollock et al. 2000; Zahm and Devesa 1995).

Until the early 1980s, leukemia was the most common cause of death due to cancer among children in the United States (Zipf et al. 2000). The mortality rate for all childhood leukemias decreased by 20% from 1975 through 1995 (Linet et al. 1999), and the rate of children dying of leukemia has significantly decreased compared with the death rate from childhood brain tumors (Bleyer et al. 1998; Ries et al. 1998).

The overall cure rate for childhood ALL is now approximately 75–80%; however, for AML the cure rate is between 40 and 45% (Pui et al. 2003). In earlier large national trials, treatment results for African American children had not been as impressive as they were for Caucasian children (Kalwinsky et al. 1985; Pinkel 1993). A recent study performed at St. Jude Children’s Hospital compared the clinical outcomes of therapy for African American and Caucasian children with ALL and concluded that with equal access to effective antileukemic therapy, children of both racial groups can expect the same high rate of ALL cure (Pui et al. 2003). However, another author concluded that ethnic or cultural differences in adherence to treatment have not been examined systematically in sufficiently large populations in the United States to draw meaningful conclusions regarding the impact of nonadherence on ethnic and racial differences in outcome of children with ALL (Bhatia 2004).

## Risk Factors

Epidemiologic studies of acute leukemias in children have examined a number of possible risk factors (e.g., environmental, genetic, or infectious) in an effort to determine the etiology of the disease. Only one environmental risk factor (ionizing radiation) has been significantly linked with either ALL or AML; most environmental risk factors [e.g., electromagnetic fields (EMFs), cigarette smoking] have been weakly or inconsistently associated with either form of childhood leukemia.

Because childhood leukemia is a rare occurrence, prospective studies are difficult to conduct, and therefore studies most frequently use a retrospective case–control design. Although easier to conduct, these studies have several inherent limitations (Schulz and Grimes 2002). For example, bias may be introduced into a retrospective study because exposure is measured indirectly, is self-reported, and may be differentially recalled by parents of a well, versus a sick, child (Infante-Rivard and Jacques 2000). Bias may also be introduced if a certain segment of the population does not respond to the study survey. Other limitations include a relatively small number of case children, the number of spurious results that arise from making many comparisons, and the questionable relevance of cross-sectional sampling after illness has developed, given the latency of the disease (Schulz and Grimes 2002). Despite these limitations, results from epidemiologic research that identifies environmental risk factors for childhood leukemia can support measures to alleviate potentially harmful exposures and reduce the risk for disease.

The environmental risk factors we discuss in this article include some of the more researched and controversial topics including ionizing and nonionizing radiation, chemicals such as hydrocarbons and pesticides, alcohol, tobacco use, and illicit drug use. Knowledge of these particular risk factors can be used to support measures to alleviate potentially harmful exposures and reduce the risk of disease. We also review genetic and infectious risk factors and other variables, including maternal reproductive history and birth characteristics.

## Environmental Factors

### Ionizing radiation

Ionizing radiation is one of the few exposures for which the causal relationship with childhood leukemia, particularly AML, has been established (Mahoney et al. 2004; Ron 1998; Sali et al. 1996; United Nations Scientific Committee on the Effects of Atomic Radiation 1994). The magnitude of the risk depends on the dose of radiation, the duration of exposure, and the age of the individual at the time of exposure. Studies have demonstrated the relationship between the degree of irradiation and occurrence of leukemia (Miller 1967; Moloney 1955). For example, leukemia rates among survivors who were within 1,000 m of the atomic bomb explosions at Hiroshima and Nagasaki, Japan, were 20-fold higher than rates among the general population (Mahoney 1955). The potential effect of ionizing radiation exposure on children may occur during preconception, pregnancy, or the postnatal period.

#### Paternal preconception exposure and close proximity to a nuclear facility

Numerous epidemiologic studies have investigated the potential association between childhood leukemia and paternal ionizing radiation exposure in the workplace before conception or preconception, also referred to as “preconception paternal irradiation” (PPI). Some studies have found an association (Gardner 1991; Gardner et al. 1990; McKinney et al. 1991; Meinert et al. 1999; Roman et al. 1993; Shu et al. 1994), whereas others have not (Doll et al. 1994; Kinlen et al. 1993; Little 1990; McLaughlin et al. 1993; Shu et al. 2002; Urquhart et al. 1991; Watson 1991).

Gardner et al. (1990) examined whether the observed excess of childhood leukemia was related to nearness to Sellafield nuclear plant in the West Cumbria district of the United Kingdom from 1950 to 1985. Relative risk was higher for leukemia in children born near Sellafield and for children of fathers employed at the plant at their conception [relative risk (RR) = 2.4; 95% confidence interval (CI), 1.04–5.7] and higher for children of fathers receiving a total preconceptual ionizing radiation dose of 100 mSv or more (RR = 6.4; 95% CI, 1.57–26.3). Gardner et al. (1990) hypothesized that irradiation of the testes may be leukomogenic in the offspring. The conclusions of this study have received significant criticism (Doll et al. 1994; Neel and Schull 1991). First, no excess leukemia was noted in the rest of Cumbria where > 90% of the births to Sellafield employees occurred (Draper et al. 1993). Second, data on the children of male atomic bomb survivors have not shown an increased incidence of leukemia (Yoshimoto et al. 1990), despite receiving a significantly greater dose of radiation than the children’s fathers at Sellafield. The results reported by Gardner et al. (1990) would suggest a much greater genetic sensitivity to radiation and therefore would be less plausible (Doll et al. 1994; Little 1990). Third, the results from the Gardner et al. (1990) study have not been substantiated by other studies that detected no statistically significant excess of leukemia in relation to nuclear facilities in the following locations:

- Scotland from 1958 to 1990 (Kinlen et al. 1993), including near the Dounreay nuclear installation in Caithness (Urquhart et al. 1991)
- Ontario, Canada, near five nuclear installations (McLaughlin et al. 1993)
- Southern England, near two sites (Roman et al. 1993), and in three areas of northern England (McKinney et al. 1991)

McKinney et al. (1991) did show a nonstatistically significant increase between childhood leukemia and reported preconceptual exposure of fathers to ionizing radiation [odds ratio (OR) = 2.35; 95% CI, 0.92–6.2]. This study overlapped with another study and the quantity of the exposure to ionizing radiation was not determined (McKinney et al. 1991).

With regard to diagnostic X rays, Shu et al. 1994) showed an increased risk for childhood leukemia related to paternal diagnostic x-ray exposure before conception in two separate studies. In the 1994 study, an increased risk was noted if two or more X rays of the lower abdomen were performed (OR = 3.8; 95% CI, 1.5–9.6). No increased risk was noted in the Shu et al. (2002) study, however, if the data were restricted to lower abdominal X rays, the more relevant area of exposure.

Mechanisms of potentiation by PPI are discussed in detail elsewhere (Lord 1999). Maternal preconception occupational exposure has not been shown to significantly increase the risk for childhood leukemia (Meinert et al. 1999; Shu et al. 1994).

#### *In utero* exposure to ionizing radiation

Many studies have attempted to ascertain the effect of *in utero* radiation exposure and the development of leukemia—particularly related to the atomic bomb exposures in Japan at the end of World War II, the 1986 Chernobyl (Russia) accident, or exposure to diagnostic X rays. An increased risk of childhood leukemia in the offspring of atomic bomb survivors has yet to be shown (Doll and Wakeford 1997; Neel 1991; Yoshimoto et al. 1988). Some studies on the Chernobyl accident, which led to widespread radioactive fallout in Europe, have found an increased risk for leukemia in children exposed *in utero* while living in areas exposed to ionizing radiation after the accident (Noshchenko et al. 2001; Petridou et al. 1996). The observed effects are in question, however, and other studies have not confirmed an increase in risk (Michaelis et al. 1997; Sali et al. 1996; Steiner et al. 1998).

In a critical review of several European studies undertaken 10 years after Chernobyl, overall, no significant association between the risk of childhood leukemia in children 0–14 years of age and exposure from the Chernobyl accident was found (Sali et al. 1996). However, a small risk could not be ruled out secondary to limited power and the fact that most studies were descriptive. In a Swedish study, the risk for ALL in children < 5 years of age in highly contaminated areas was increased although not statistically significant (OR = 1.5; 95% CI, 0.8–2.6) (Hjalmars et al. 1994). Last, in a review of 36 cancer registries in Europe for children 0–14 years of age, the risk for leukemia was increased slightly in the postaccident period, but the overall geographical patterns of change uncovered no relation to the estimated exposure to radiation (Parkin et al. 1993, 1996).

Many studies have assessed the risk of *in utero* exposure to diagnostic X rays and the development of childhood leukemia, with conflicting results. The potential risk of leukemia in children exposed to diagnostic X rays was first noted in 1956 (Stewart et al. 1956). Many case–control studies have found a consistent association to suggest that radiographic examination of the abdomen of a pregnant woman produces a proportional increase in risk of about 40% (Doll and Wakeford 1997). The evidence for causality and for uncertainty is well described (Boice and Miller 1999; Doll and Wakeford 1997). The most notable reason for doubt of a true association is the lack of evidence in cohort studies for an increased risk of leukemia in children exposed to radiation *in utero* during the atomic bomb blasts in Japan. Furthermore, several recent studies have not found a significant association between childhood leukemia and X-ray examinations in the mother during pregnancy (Meinert et al. 1999; Naumberg et al. 2001; Shu et al. 2002). This apparent decrease in risk over time may be attributable to declining exposures to ionizing radiation (decreased dose) and to the increasing use of diagnostic ultrasound in place of diagnostic X rays during pregnancy (Shu et al. 2002).

#### Postnatal exposure to ionizing radiation

Postnatal exposure to ionizing radiation has been demonstrated to increase the risk of childhood leukemia, most notably from the World War II atomic bomb blasts and radiotherapy for benign disease. Children and adolescents exposed to 1 gray after the bombings were at elevated risk for leukemia (RR = 7.1) and developed leukemia in less time when compared with adults (Boice 1996). Several studies have identified an increased risk for childhood leukemia or death due to leukemia secondary to radiotherapy of benign disease (Darby et al. 1987; Davies et al. 1961; Murray et al. 1959; Ron et al. 1988) whereas others have not (Lundell and Holm 1996) (Table 1). This risk appears to be associated with therapeutic radiation for thymus enlargement, ankylosing spondylitis, and ringworm.

Although inconclusive, to date, radiation exposure secondary to the Chernobyl accident has not been shown to increase the risk of leukemia in children who were exposed after birth (Moysich et al. 2002; Parkin et al. 1993, 1996; Sali et al. 1996). Recent studies have shown mixed results regarding exposure to diagnostic X rays during the postnatal period and the risk for childhood leukemia (Meinert et al. 1999; Shu et al. 2002) (Table 1). Shu et al. (2002) found no significant risk for ALL, even if there were exposure to three or more rays (OR = 1.2; 95% CI, 1.0–1.6). They did note a significant risk for children with pre-B cell ALL, most notably if three or more X rays were performed (OR = 3.2; 95% CI, 1.5–7.2) and if the child was older than 5 years (OR = 3.8; 95% CI, 1.1–13.3). Meinert et al. (1999) found that exposure to four or more X rays in the postnatal period did not contribute a significant risk to childhood leukemia (OR = 2.3; 95% CI, 0.8–6.5); however, the risk was not evaluated by immunophenotype. Overall, postnatal X-ray studies do not appear to significantly increase the risk of childhood leukemia. Increased risk for specific immunophenotypes may result from certain biases, but this may be an area for further study.

### Nonionizing radiation

Numerous epidemiologic studies have been conducted to determine whether an association exists between exposure to nonionizing EMFs and childhood leukemia; some have found a small association (Ahlbom et al. 2000; Greenland et al. 2000; Hatch et al. 1998; Rivard and Deadman 2003; Savitz et al. 1990) while others have not (Kleinerman et al. 2000; Linet et al. 1997; Myers et al. 1990). The inconsistent results of the EMF and childhood leukemia studies may be due in part to differing methods for assessing residential magnetic field exposures and unmeasured EMF characteristics (Bowman and Thomas 2001; Brain et al. 2003; Hardell et al. 1995). Furthermore, investigations of animals with exposure to much higher levels of EMFs than humans have not shown increased risk for hematopoietic neoplasia (Brain et al. 2003).

Ahlbom et al. (2000) conducted a pooled analysis of data collected for nine studies. For the 44 children with leukemia and 62 control children with high estimated residential EMF exposure, the estimated summary relative risk was 2.00 (95% CI, 1.3–3.1). Adjusting for potential confounding variables did not appreciably change the results, but selection bias may have accounted for some of the increase. Linet et al. (1997) conducted a case–control study with actual measurements of EMFs in the homes of > 1,200 study subjects near the time of the diagnosis of ALL. Overall, there was no statistically significant increased risk of ALL with increasing exposure to residential magnetic fields (OR = 1.24; 95% CI, 0.86–1.79) for the highest exposure category compared with the lowest. Furthermore, no increased risk of ALL was found based on EMF levels for homes in which the mother resided during pregnancy with the index child (Linet et al. 1997). For additional information on nonionizing radiation, see Supplemental Material (http://www.ehponline.org/docs/2006/9023/abstract.html).

### Chemicals

The chemical classes most commonly associated with childhood leukemia are hydrocarbons and pesticides. Studies have examined the relationship between childhood leukemia and direct exposure to these chemicals (e.g., use of pesticides in the home) (Freedman et al. 2001; Lowengart 1987) as well as secondary exposure, such as to parents’ clothing worn in an occupational setting where hydrocarbons are used and brought into the home to be laundered (Buckley et al. 1989; Lowengart 1987; Shu et al. 1999b).

#### Hydrocarbons

Hydrocarbons are organic compounds made up primarily of carbon and hydrogen atoms. Examples of hydrocarbon compounds include gasoline and trichloroethylene (spot remover). Hydrocarbons are found in many household and industrial products including paint removers and thinners, and solvents, which are used to dissolve other chemical substances.

The most widely recognized hydrocarbon is benzene, a ubiquitous chemical used in the manufacture of paints and plastics and as a constituent in motor fuels and hobby glues. It is also formed during incomplete combustion of fossil fuels (i.e., petroleum products, coal). Benzene is a known human carcinogen. It has a strong positive exposure–response relationship with leukemia, particularly AML, at a range of exposures not much higher than the current legal standard for workers as recommended by the National Institute for Occupational Safety and Health (Rinsky 1981). A recent occupational study (Glass 2003) found excess risk for leukemia associated with cumulative benzene exposures and benzene exposure intensities at lower levels (< 60 ppm-years) than had been previously reported (as high as 220 ppm-years) (Rushton and Romaniuk 1997; Schnatter et al. 1996). For additional information on benzene, see Supplemental Material (http://www.ehponline.org/docs/2006/9023/abstract.html).

Freedman et al. (2001) conducted a case-control study of 640 subjects to examine the relationship between parental hobbies and home projects and incidence of childhood leukemia. ALL had a statistically significant association with prenatal exposure to painted homes (> 4 rooms) (OR = 1.7; 95% CI, 1.1–2.7) and to artwork with solvents (OR = 4.1; 95% CI, 1.1–15.1). However, the study was limited by the lack of information about the child’s proximity to the activity and the timeframe of exposure.

In a review by Savitz and Chen (1990) of seven published epidemiologic studies of the association between parental occupation and childhood leukemia, exposure to paints and pigments yielded the most consistently positive results, with several studies producing ORs for ALL and AML of ≥ 1.5. Paternal occupations most associated with increased risk for childhood leukemia in the review included painter, motor vehicle–related worker and machinist or factory worker. In one of the seven epidemiologic studies reviewed, paternal hydrocarbon exposures (e.g., gas station attendant) appeared to be related to both AML and ALL occurring in children ≤ 1 year of age (Vianna et al. 1984), but this finding could not be confirmed by other studies that covered a broader age range (Van Steensel-Moll et al. 1985).

In a separate large case–control study, only paternal long-term exposure to plastics during the preconception period showed an increased risk of childhood leukemia, though not statistically significant (OR = 1.4; 95% CI, 1.0–1.9) (Shu et al. 1999b).

There is much speculation about the biological mechanism by which paternal occupational exposures may affect the offspring’s risk for leukemia. Potential mechanisms include genetic alteration of the father’s sperm, which may transmit cancer susceptibility to the child, transplacental fetal exposure after the father brings a toxic exposure into the home, and direct postnatal exposure to chemicals brought home on the clothing (e.g., off-gassing) (Buckley et al. 1989; Savitz and Chen 1990) or indirect exposure through breast-feeding. Exposure through breast milk, however, is an unlikely mechanism as breast-feeding has been associated with a reduced risk of ALL (Shu et al. 1999a). The reduction in risk was stronger with a longer duration of breast-feeding.

Specific occupations found more frequently among mothers of children with leukemia include metal manufacturing or processing, textiles, and pharmacist (Savitz and Chen 1990; Van Steensel-Moll et al. 1985). Increased risk for ALL has been described, in a case–control study of more than 3500 children, with maternal occupational exposure to solvents (OR = 1.8; 95% CI, 1.3–2.5) and paints (OR = 1.6; 95% CI, 1.2–2.2) during the preconception period (OR = 1.6; 95% CI, 1.3–2.5), and during pregnancy (OR = 1.7; 95% CI, 1.2–2.3), but not during the postnatal period (Shu et al. 1999b). Despite these associations, no linear dose–response relationship was observed in this study for any maternal exposure during any time period, and statistically significant associations were found only when maternal occupational exposures were short (equal or below the median) (Shu et al. 1999b).

A recent study in California suggested that children living in areas with high levels of point source carcinogenic hazardous air pollutants (HAPs) are at an increased risk of developing leukemia (RR = 1.3; 95% CI, 1.13–1.6) (Reynolds et al. 2003). Selected carcinogenic HAPs included benzene, perchloroethylene, and trichloroethylene. This study had several limitations; most notably the extrapolation of group exposure levels to individuals and exclusion of indoor HAP sources (e.g., tobacco smoke) as potential confounders of exposure estimates (Reynolds et al. 2003).

A recent, similar study in Great Britain reported associations between birthplace of children with leukemia and proximity to industrial sites that release volatile organic compounds, dioxins, 1,3-butadiene, and benzo[*a*]pyrene (Knox 2005).

#### Pesticides

Many studies have suggested a link between pesticide exposure and childhood leukemia. However, most of these studies are limited by the use of nonspecific pesticide exposure information, small numbers of exposed children, and potential recall bias. Some studies suggest that pesticide-exposed fetuses and children are at higher risks for cancer compared with adults. This suggests that newborns and children may be particularly sensitive to the carcinogenic effects of pesticides (National Research Council 1993; Zahm and Ward 1998). Most children’s exposure to pesticides is from home, lawn, and garden use (Grossman 1995). Other sources of exposure can include local agricultural applications, contaminated food, parental occupation, and pet products.

In a large meta-analysis of epidemiologic studies that investigated whether occupational or residential exposure to pesticides by either parents or children was related to increased risk of childhood cancer, frequent occupational exposure or in-home pesticide use was associated with childhood leukemia (Daniels et al. 1997) (Table 2). Use of garden pesticides or professional exterminations did not greatly affect risk in this meta-analysis. Some studies have reported increased risk with mothers who had frequent prenatal pesticide exposure in the garden (Infante-Rivard et al. 1999) or during pregnancy (OR = 6.5; 95% CI, 1.5–59) (Lowengart 1987) and increased risk during childhood with garden insecticides (OR = 2.4; 95% CI, 1.3–4.3) and garden fungicides (OR = 2.5; 95% CI, 1.0–6.2) (Menegaux et al. 2006).

Children exposed to professional pest control services at any time 1 year before birth to 3 years after had increased risk as well (OR = 2.8; 95% CI, 1.4–5.7) (Ma 2002) (Table 2). In a review of 18 studies that assessed the association between pesticide exposure and childhood cancer, no clear patterns of risk by which parent was exposed, by timing of exposure, or by histologic type of leukemia were apparent (Zahm and Ward 1998).

In a recent case–control study conducted in France, for the first time insecticidal shampoo treatment of pediculosis was found to be associated with childhood ALL and AML (OR = 1.9; 95% CI, 1.2–3.3) (Menegaux et al. 2006). Various insecticidal shampoos were reported but only the association with pyrethroid-based shampoo was statistically significant (OR = 2.0; 95% CI, 1.1–3.4) (Menegaux et al. 2006).

### Alcohol, cigarette, and illicit drug use

Studies have shown an increased risk for AML, particularly among very young children, associated with maternal alcohol consumption during pregnancy (Shu et al. 1996; Van Duijn et al. 1994). Shu et al. (1996) examined the effect of alcohol consumption on the risk of developing childhood leukemia, beginning 1 month before and continuing throughout the pregnancy. The risk for AML with maternal alcohol consumption (OR = 2.6; 95% CI, 1.4–5.1) was almost twice that of ALL. Paternal alcohol consumption before conception did not appear to increase risk (Shu et al. 1996).

It is unclear whether maternal or paternal cigarette smoking before or during pregnancy is a risk factor for developing childhood leukemia (Brondum et al. 1999; Shu et al. 1996). Two studies found that the frequency, amount, and duration of paternal smoking before conception were related to significantly elevated risk, after adjusting for the effect of maternal smoking (Ji et al. 1997; Sorahan et al. 1995). Brondum et al. (1999), however, found no association between the disease and paternal smoking at any time.

Maternal marijuana use before and during pregnancy has been associated with childhood AML and ALL. Despite a small sample size, findings from a Children’s Cancer Group study showed a 10-fold risk of childhood AML associated with maternal use of marijuana just before or during pregnancy (Robison et al. 1989). The authors concluded that, because marijuana has been shown to be a teratogen in animals and possibly humans, it may be leukomogenic, either alone or in combination with a cofactor.

## Genetics

Childhood leukemia and other cancers may stem from a combination of genetic susceptibility factors and environmental exposures. The importance of *in utero* genetic events has been suspected for many years based on concordance studies on twins with leukemia (Clarkson and Boyse 1971; Greaves et al. 2003; Zipf et al. 2000). This is especially true among identical twins. An identical twin is twice as likely as the general population to develop leukemia if his or her twin developed the illness before the age of 7 years (Miller 1967; Zipf et al. 2000). Twins who reach age 15 years without developing leukemia do not appear to be at higher risk of developing the disease.

This concept that some cases of childhood leukemia originate *in utero* also is the result of genetic studies that have found leukomogenic translocations or clonotypic gene fusion sequences that match that of later leukemic blasts in heelstick blood samples (Guthrie cards) from newborns who later developed ALL (Gale et al. 1997; Greaves et al. 2003). Infant acute leukemia commonly involves gene fusions with the MLL gene. Because (MLL+) acute leukemia can arise after chemotherapy with DNAt2 inhibitors, it is possible that these substances, which are found naturally in certain foods and beverages (e.g., fruits and vegetables, legumes, coffee), may contribute toward infant leukemia (Alexander et al. 2001; Ross et al. 1996; Spector et al. 2005).

Studies of archived neonatal blood spots in which a specific gene fusion occurs [t(1;19)/E2A-PBX1] support the theory that not all cases of childhood ALL develop *in utero* (Wiemels et al. 2002). The association of leukemogenesis in children with metabolizing gene variants suggests a causal relationship to environmental exposures that may occur during the postnatal period. Increased risk of childhood leukemia has recently been associated with genetic polymorphisms that disrupt the hosts’ abilities to properly metabolize and transport xenobiotic exposures (Canalle et al. 2004; Infante-Rivard and Jacques 2000; Krajinovic et al. 2002). Furthermore, polymorphic genes encoding carcinogen- and drug-metabolizing enzymes may not only increase the risk of ALL but also influence the risk of relapse in patients and therefore help to predict disease outcome. For example, the prognosis of patients with cytochrome P450 1A1 (*CYP1A1*) and NAD(P)H quinine oxi-doreductase 1 (*NQO1*) variants has been found to be worse than that of patients who lack these variants (Krajinovic et al. 2001).

Although genetic syndromes account for only a small proportion of childhood leukemias, certain inherited diseases are associated with a higher risk of developing leukemia. Examples of these diseases include Fanconi anemia (Swift 1971; Willis and Lindahl 1987), Bloom syndrome (Miller 1968; Willis and Lindahl 1987), ataxia telangiectasia (Toledano and Lange 1980), Down syndrome (Dordelmann et al. 1998; Robison et al. 1987), Shwachman syndrome (Woods et al. 1981), and neurofibromatosis (Shearer et al. 1994). These inherited diseases are characterized by defective DNA repair, chromosome aneuploidy (an abnormal number of chromosomes), or chromosomal abnormalities such as translocations. AML has a higher incidence during the neonatal period than ALL among children with Fanconi anemia, Bloom syndrome, and Down syndrome.

Siblings of children with leukemia are at greater risk of developing leukemia than children whose siblings do not have the disease (Draper et al. 1977; Heath and Moloney 1965). Also, a positive family history of hematopoietic malignancies among first- or second-degree relatives has been associated with a small increased risk for childhood ALL. Furthermore, the risk for ALL is not increased in children with a family history of cancers other than hematopoietic malignancies (Infante-Rivard and Guiguet 2004).

## Infectious Agents and the Population Mixing Theory

Several observations contribute to the theory that a transmissible agent is potentially involved in the oncogenic process of childhood leukemia. First, the peak incidence of childhood leukemia and that of common childhood infections both occur among children 2–5 years of age, the age group least likely to possess sophisticated immune systems (Greaves 2002; Pierce 1936). Second, a viral etiology has been shown for some animal and human cancers (e.g., Epstein-Barr virus for Burkitt lymphoma) (Greaves and Alexander 1993; Gross 1978). Third, evidence exists of an apparent seasonal variation in the birth or onset dates of childhood leukemia. Statistically significant seasonal variation for ALL with a peak in the summer (Ross et al. 1999; Westerbeek et al. 1998), in the autumn–winter (Karimi et al. 2003) and in the early spring among 1- to 6-year-old children (Feltbower et al. 2001) have been described. However, a large study of > 15,000 cases of childhood leukemia born and diagnosed in the United Kingdom found no evidence of seasonality in either month of birth or month of diagnosis in any subgroup of childhood cancer (Higgins et al. 2001). Last, an excess of childhood leukemia has been found in rural, potentially immunologically naïve, populations that have undergone an influx of permanent residents; this theory is known as population mixing (Kinlen 1988, 1995). Kinlen (1988) suggested that an extremely infectious but not highly pathogenic agent introduced into a previously unexposed community might cause an epidemic of ALL. Susceptible individuals who live in rural areas in which the population suddenly increases, or for which the composition changes regularly, may be exposed to infectious agents that are brought into the area by new residents, possibly triggering a cluster of cases of ALL. The theory implies that the differences in rates of leukemia in rural areas are due to differences in herd, or population, immunity. Results from other studies support this theory (Alexander et al. 1997; Boutou et al. 2002; Gilman and Knox 1998; Kinlen 1995; Kinlen et al. 1991; Koushik et al. 2001). For additional information on population mixing, see Supplemental Material (http://www.ehponline.org/docs/2006/9023/abstract.html).

An alternative hypothesis to Kinlen’s suggests that the mechanism of “delayed infection” is responsible, and that an abnormal response to the infection promotes a second event critical for the development of ALL (Greaves and Alexander 1993). Support of this hypothesis comes from studies showing that children with ALL are less likely to have had common infections, perhaps as a result of immunologic isolation early in life (Ma 2002). Proposed mechanisms for an abnormal response to an as yet unidentified infectious agent might include leukemogenicity of the virus itself, which may directly infect and transform B-precursor cells, or physical characteristics of the virus that stimulate critical receptors for cell growth (Greaves and Alexander 1993).

## Other Important Variables

### Maternal reproductive history and birth characteristics

Conflicting results have been reported from studies of the relationship between maternal history of fetal loss and risk for ALL or AML (Kaye et al. 1991; Ross et al. 1997; Yeazel 1995). A history of fetal loss may reflect genetic predisposition, abnormal intrauterine environment, or the effect of a common environmental exposure (Ross et al. 1997).

The risk for childhood ALL has been shown to be significantly higher among children who were born when their parents were older; significant trends in ALL incidence have been related to increasing mother’s (> 35 years, *p* < 0.001) and father’s (> 40 years, *p* < 0.002) ages (Dockerty et al. 2001). For children born to mothers and fathers ≥ 40 years of age, the odds ratio for offspring developing childhood leukemia was 1.97 and 1.45, respectively (Dockerty et al. 2001).

Several studies have described an association between increased birth weight and childhood ALL, particularly ALL developing early in life (Kaye et al. 1991; Robison et al. 1987; Ross et al. 1997). Increased birth weight has been associated with a high rate of cell proliferation and with an increase in the number of precursor cells that are at risk for malignant transformation (Westergaard 1997). However, birth weight tends to increase with maternal age, which is also a known risk factor for ALL in offspring (Dockerty et al. 2001).

For information on medications/therapeutic agents, military history and nutrition in relation to childhood leukemia, see Supplemental Material (http://www.ehponline.org/docs/2006/9023/abstract.html).

## Conclusion

ALL and AML are the most common malignancies of childhood. Despite many advances in the treatment of childhood leukemia, the causative factors of ALL and AML remain unclear. Identifying risk factors for childhood leukemia (e.g., environmental, genetic, infectious) is an important step in the reduction of the overall burden of the disease. In general, benzene and ionizing radiation are two environmental exposures strongly associated with the development of childhood AML or ALL. However, identifying other environmental risk factors associated with leukemia is difficult for several reasons:

- inability to confirm and quantify exposures;
- lack of a prospective cohort;
- presence of different variants of leukemia; presence of confounders; and
- inadequate understanding of the pathophysiology of leukemia (e.g., gene–environment or infection–environment interaction).

Future attempts to overcome these obstacles (e.g., through improving laboratory detection methods to confirm exposure; prospective cohort studies; focusing on specific types of leukemia and specific chemical agents) could possibly uncover other environmental risk factors associated with childhood leukemia and mitigate potentially harmful exposures to reduce the risk for disease. Future studies, when appropriate, should attempt to use common questionnaires, address timing and route of exposure, document evidence that exposure has actually been transferred from the workplace to the child, and store biological samples when possible.

## Correction

In the Abstract and in the section “Risk Factors,” the sentences “Only two environmental risk factors (benzene and ionizing radiation) have been significantly linked to ALL or AML” in the original manuscript published online have been changed here to “Only one environmental risk factor (ionizing radiation) has been significantly linked to ALL or AML.”

## Figures and Tables

**Table 1:** Postnatal ionizing radiation exposure from diagnostic X rays and radiotherapy and the risk of childhood leukemia.

Reference	Study design	Study question	No.	Results	RR or OR (95% CI)
Diagnostic X rays					
Shu et al. 2002	Retrospective case control	Risk of ALL from exposure to diagnostic X rays	3,828	No increased risk for any X rays done	OR 1.1 (0.9–1.2)
				No increased risk for ≥ 3 X rays	OR 1.2 (1.0–1.6)
				Increase risk for pre-B cell ALL	OR 3.2, (1.5–7.2)
Meinert et al. 1999	Retrospective case control	Risk of leukemia in children from exposure to diagnostic X rays	437	No significant increase in risk if exposed to ≥ 4 X rays	OR 2.3 (0.8–6.5)
Radiotherapy					
Murray et al. 1959	Retrospective case control	Risk of leukemia in children exposed to ionizing radiation for thymic enlargement	2,750	Greater than expected numbers of leukemia deaths if treated for thymic enlargement	Ratio of observed to expected deaths = 4.5
Darby et al. 1987^a^	Prospective	Risk of cancer in children who received radiotherapy for ankylosing spondylitis	> 14,000	Increase in leukemia mortality in children treated with radiotherapy for ankylosing spondylitis	3-fold increased risk
Lundell and Holm 1996	Retrospective cohort	Increased risk of mortality from leukemia after irradiation for skin hemangioma	> 14,000	No excess risk of mortality from leukemia	RR greatest for AML
				No significant association between childhood leukemia and radiation dose	RR = 3.2 from dose of > 0.01–0.10 to > 0.10 gray (95% CI, 1.0–7.4)

a The observed death rate for children with leukemia increased over the first 5 years of observation, then gradually decreased

**Table 2:** Pesticide exposure and the risk of childhood leukemia.

Reference	Study design	Study question	No.	Results	RR or OR (95% CI)
Lowengart 1987	Retrospective case control	Association between childhood leukemia and occupational and home exposures	> 225	Increased risk for household use of pesticides by either parent during pregnancy	OR 3.8 (1.4–13)
Buckley et al. 1989	Retrospective case control	Risk of AML as a result of parental occupational exposure to pesticides	> 400	Increased risk for AML in all children for whom paternal pesticide exposure in jobs were held > 1,000 days	OR 2.7 (1.0–7.0)
				Increased risk for AML in children < 5 years of age for whom paternal pesticide exposure in jobs were held > 1,000 days	OR 11.4 (1.5–88.7)
Leiss and Savitz 1995	Retrospective case control	Association between childhood cancer and home pesticide use	> 450	Association between childhood leukemia and use of no-pest strips (contain dichlorvos)	OR 1.7–3.0 ( > 1) Highest for exposure during the last 3 months of pregnancy
Meinert et al. 1996	Retrospective case control	Association between childhood leukemia and pesticide exposure	> 400	Increased risk of leukemia in children whose parents used pesticides in a garden from 2 years before birth to date of diagnosis	OR 2.5 (1.0–6.1)
Daniels et al. 1997	Meta-analysis	Association between childhood cancer and pesticide exposure	31 studies	For leukemia, five of nine occupational studies showed a positive association	Not applicable
Ma et al. 2002b	Prospective case control	Risk of childhood leukemia and household pesticide use	> 300	Increased risk with use of professional pest control services	OR 2.8 (1.4–5.7)
				No significant association for exposure to outdoor pesticides	
Reynolds et al. 2002	Retrospective cohort	Risk of childhood cancer and agricultural pesticide use	> 2,000	Childhood leukemia rates elevated in areas with higher use of propargite, but no dose–response trend was noted	RR 1.48 (1.03–2.13)

## References

[b1-ehp0115-000138] Ahlbom A, Day N, Feychting M, Roman E, Skinner J, Dockerty J (2000). A pooled analysis of magnetic fields and childhood leukaemia. Br J Cancer.

[b2-ehp0115-000138] Alexander FE, Chan LC, Lam TH, Yuen P, Leung NK, Ha SY (1997). Clustering of childhood leukaemia in Hong Kong: association with the childhood peak and common acute lymphoblastic leukaemia and with population mixing. Br J Cancer.

[b3-ehp0115-000138] Alexander FE, Patheal SL, Biondi A, Brandalise S, Cabrera ME, Chan LC (2001). Transplacental chemical exposure and risk of infant leukemia with MLL gene fusion. Cancer Research.

[b4-ehp0115-000138] Bhatia S (2004). Influence of race and socioeconomic status on outcome of children treated for childhood acute lymphoblastic leukemia. Curr Opin Pediatr.

[b5-ehp0115-000138] Bleyer A, Sposto R, Sather H (1998). In the United States, pediatric brain tumors and other nervous system tumors are now more common than childhood acute lymphoblastic leukemia (ALL) and have a 3-fold greater national mortality rate than ALL [Abstract]. Proc Am Soc Clin Oncol.

[b6-ehp0115-000138] Boice J (1996). Cancer following irradiation in childhood and adolescence. Med Pediatr Onc (suppl).

[b7-ehp0115-000138] Boice J, Miller R (1999). Childhood and adult cancer after intrauterine exposure to ionizing radiation. Teratology.

[b8-ehp0115-000138] Boutou O, Guizard A-V, Slama R, Pottier D, Spira A (2002). Population mixing and leukaemia in young people around the La Hague nuclear waste reprocessing plant. Br J Cancer.

[b9-ehp0115-000138] Bowman JD, Thomas DC (2001). Re: “Are children living near high-voltage power lines at increased risk of acute lymphoblastic leukemia?. Am J Epidemiol.

[b10-ehp0115-000138] Brain JD, Kavet R, McCormick DL, Poole C, Silverman LB, Smith TJ (2003). Childhood leukemia: electric and magnetic fields (EMF) as possible risk factors. Environ Health Perspect.

[b11-ehp0115-000138] Brondum J, Shu XO, Steinbuch M, Severson RK, Potter JD, Robison LL (1999). Parental cigarette smoking and the risk of acute leukemia in children. Cancer.

[b12-ehp0115-000138] Buckley JD, Robison LL, Swotinsky R, Garabrant DH, LeBeau M, Manchester P (1989). Occupational exposures of parents of children with acute nonlymphoblastic leukemia: a report from the Children’s Cancer Study Group. Cancer Res.

[b13-ehp0115-000138] Canalle R, Burim RV, Tone LG, Takahashi CS (2004). Genetic polymorphisms and susceptibility to childhood acute lymphoblastic leukemia. Environ Mol Mutagen.

[b14-ehp0115-000138] Clarkson B, Boyse EA (1971). Possible explanation of high concordance for acute leukemia in monozygous twins. Lancet.

[b15-ehp0115-000138] Daniels JL, Olshan AF, Savitz DA (1997). Pesticides and childhood cancers. Environ Health Perspect.

[b16-ehp0115-000138] Darby SC, Doll R, Gill SK, Smith PG (1987). Long term mortality after a single treatment course with X-rays in patients treated for ankylosing spondylitis. Br J Cancer.

[b17-ehp0115-000138] Davies A, Modan B, Djaldetti M, deVries A (1961). Epidemiological observations on leukemia in Israel. Arch Intern Med.

[b18-ehp0115-000138] Dockerty JD, Draper G, Vincent T, Rowan SD, Bunch KJ (2001). Case-control study of parental age, parity and socioeconomic level in relationship to childhood cancers. Int J Epidemiol.

[b19-ehp0115-000138] Doll R, Evans HJ, Darby SC (1994). Paternal exposure not to blame. Nature.

[b20-ehp0115-000138] Doll R, Wakeford R (1997). Risk of childhood cancer from fetal irradiation. Br J Radiol.

[b21-ehp0115-000138] Dordelmann M, Schrappe M, Reter A, Zimmerman M, Graf N, Schott G (1998). Down’s syndrome in childhood acute lymphoblastic leukemia: clinical characteristics and and treatment outcome in four consecutive BFM trials, Berlin-Frankfurt-Munster Group. Leukemia.

[b22-ehp0115-000138] Draper GJ, Heaf MM, Kinnier Wilson LM (1977). Occurrence of childhood cancers among sibs and estimation of familial risks. J Med Genet.

[b23-ehp0115-000138] Draper GJ, Stiller CA, Cartwright RA, Craft AW, Vincent TJ (1993). Cancer in Cumbria and in the vicinity of the Sellafield nuclear installation, 1963–1990. BMJ.

[b24-ehp0115-000138] Feltbower RG, Pearce MS, Dickinson HO, Parker L, McKinney PA (2001). Seasonality of birth for cancer in Northern England, UK. Paediatr Perinat Epidemiol.

[b25-ehp0115-000138] Freedman MD, Stewart P, Kleinerman RA, Wacholder S, Hatch EE, Tarone RE (2001). Household solvent exposures and childhood acute lymphoblastic leukemia. Am J Public Health.

[b26-ehp0115-000138] Gale KB, Ford AM, Repp R, Borkhardt A, Keller C, Eden OB (1997). Backtracking leukemia to birth: identification of clonotypic gene fusion sequences in neonatal blood spots. Proc Natl Acad Sci USA.

[b27-ehp0115-000138] Gardner MJ (1991). Father’s occupational exposure to radiation and the raised level of childhood leukemia near the Sellafield Nuclear Plant. Environ Health Perspect.

[b28-ehp0115-000138] Gardner MJ, Snee MP, Hall AJ, Powell CA, Downes S, Terrell JD (1990). Results of case-control study of leukemia and lymphoma among young people near Sellafield nuclear plant in West Cumbria. BMJ.

[b29-ehp0115-000138] Gilman EA, Knox EG (1998). Geographical distribution of birth places of children with cancer in the UK. Br J Cancer.

[b30-ehp0115-000138] Glass DC (2003). Leukemia risk associated with low-level benzene exposure. Epidemiology.

[b31-ehp0115-000138] Greaves MF (2002). Childhood leukemia. BMJ.

[b32-ehp0115-000138] Greaves MF, Alexander FE (1993). An infectious etiology for common acute lymphoblastic leukemia in childhood?. Leukemia.

[b33-ehp0115-000138] Greaves MF, Maia AT, Wiemels JL, Ford AM (2003). Leukemia in twins: lessons in natural history. Blood.

[b34-ehp0115-000138] Greenland S, Sheppard AR, Kaune WT, Poole C, Kelsh MA (2000). A pooled analysis of magnetic fields, wire codes, and childhood leukemia, Childhood-EMF Study Group. Epidemiology.

[b35-ehp0115-000138] Greenlee RT, Murray T, Bolden S, Wingo PA (2000). Cancer statistics 2000. CA Cancer J Clin.

[b36-ehp0115-000138] Gross L (1978). Viral etiology of cancer and leukemia: a look into the past, present and future. Cancer Res.

[b37-ehp0115-000138] Grossman J (1995). What’s hiding under the sink: dangers of household pesticides. Environ Health Perspect.

[b38-ehp0115-000138] Gurney JG, Severson RK, Davis S, Robison LL (1995). Incidence of cancer in children in the United States, sex, race, and 1-year age-specific rates by histologic type. Cancer.

[b39-ehp0115-000138] Hardell L, Holmberg B, Malker H, Paulsson LE (1995). Exposure to extremely low frequency electromagnetic fields and the risk of malignant disease—an evaluation of epidemiological and experimental findings. Eur J Cancer Prev.

[b40-ehp0115-000138] Hatch EE, Linet MS, Kleinerman RA, Tarone RE, Severson RK, Hartsock CT (1998). Association between childhood acute lymphoblastic leukemia and use of electrical appliances during pregnancy and childhood. Epidemiology.

[b41-ehp0115-000138] Heath CW, Moloney WC (1965). Familial leukemia: five cases of acute leukemia in three generations. N Engl J Med.

[b42-ehp0115-000138] Higgins CD, dos-Santos-Silva I, Stiller CA, Swerdlow AJ (2001). Season of birth and diagnosis of children with leukaemia: an analysis of over 15000 UK cases occurring from 1953–95. Br J Cancer.

[b43-ehp0115-000138] Hjalmars U, Kulldorff M, Gustafsson G (1994). Risk of acute childhood leukemia in Sweden after the Chernobyl reactor accident. BMJ.

[b44-ehp0115-000138] Infante-Rivard C, Guiguet M (2004). Family history of hematopoietic and other cancers in children with acute lymphoblastic leukemia. Cancer Detect Prev.

[b45-ehp0115-000138] Infante-Rivard C, Jacques L (2000). Empirical study of parental recall bias. Am J Epidemiol.

[b46-ehp0115-000138] Infante-Rivard C, Labuda D, Krajinovic M, Sinnett D (1999). Risk of childhood leukemia associated with exposure to pesticides and with gene polymorphisms. Epidemiology.

[b47-ehp0115-000138] Ji BT, Shu XO, Linet M, Xheng W, Wacholder S, Gao YT (1997). Paternal cigarette smoking and risk for childhood cancer among offspring of nonsmoking mothers. J Natl Cancer Inst.

[b48-ehp0115-000138] Kalwinsky DK, Rivera G, Dahl GV, Roberson P, George S, Murphy SB (1985). Variation by race in presenting clinical and biologic features of childhood acute lymphoblastic leukemia: implications for treatment outcome. Leuk Res.

[b49-ehp0115-000138] Karimi M, Yarmohammadi H (2003). Seasonal variations in the onset of childhood leukemia/lymphoma: April 1996 to March 2000, Shiraz, Iran. Hematol Oncol.

[b50-ehp0115-000138] Kaye SA, Robison LL, Smithson WA, Gunderson P, King FL, Neglia JP (1991). Maternal reproductive history and birth characteristics in childhood acute lymphoblastic leukemia. Cancer.

[b51-ehp0115-000138] Kersey JH, Sabad A, Gajl-Peczalska K, Hallgren HM, Yunis EJ, Nesbit ME (1973). Acute lymphoblastic leukemia cells with T (thymus-derived) lymphocyte markers. Science.

[b52-ehp0115-000138] Kinlen LJ (1988). Evidence for an infective cause of childhood leukaemia: comparison of a Scottish new town with nuclear reprocessing sites in Britain. Lancet.

[b53-ehp0115-000138] Kinlen LJ (1995). Epidemiologic evidence for an infective basis in childhood leukaemia. Br J Cancer.

[b54-ehp0115-000138] Kinlen LJ, Clarke K, Balkwill A (1993). Paternal preconceptional radiation exposure in the nuclear industry and leukaemia and non-Hodgkin’s lymphoma in young people in Scotland. BMJ.

[b55-ehp0115-000138] Kinlen LJ, Hudson C, Stiller LA (1991). Contacts between adults as evidence for an infective origin of childhood leukemia: an explanation for the excess near nuclear establishments in West Berkshire?. Br J Cancer.

[b56-ehp0115-000138] Kleinerman RA, Kaune WT, Hatch EE, Wacholder S, Linet MS, Robison LL (2000). Are children living near high-voltage power lines at increased risk of acute lymphoblastic leukemia?. Am J Epidemiol.

[b57-ehp0115-000138] Knox EG (2005). Childhood cancers and atmospheric carcinogens. J Epidemiol Community Health.

[b58-ehp0115-000138] Koushik A, King WD, McLaughlin JR (2001). An ecologic study of childhood and population mixing in Ontario, Canada. Cancer Causes Control.

[b59-ehp0115-000138] Krajinovic M, Labuda D, Sinnett D (2001). Childhood acute lymphoblastic leukemia: genetic determinants of susceptibility and disease outcome. Rev Environ Health.

[b60-ehp0115-000138] Krajinovic M, Labuda D, Sinnett D (2002). Glutathione *S*-trans-ferase P1 genetic polymorphisms and susceptibility to childhood acute lymphoblastic leukaemia. Pharmacogenetics.

[b61-ehp0115-000138] Leiss JK, Savitz DA (1995). Home pesticide use and childhood cancer: a case-control study. Am J Public Health.

[b62-ehp0115-000138] Linet MS, Hatch EE, Kleinerman RA, Robison LL, Kaune WT, Friedman DR (1997). Residential exposure to magnetic fields and acute lymphoblastic leukemia in children. N Engl J Med.

[b63-ehp0115-000138] Linet MS, Ries LA, Smith MA, Tarone RE, Devesa SS (1999). Cancer surveillance series: recent trends in childhood cancer incidence and mortality in the United States. J Natl Cancer Inst.

[b64-ehp0115-000138] Little MP (1990). A comparison between the risk of childhood leukemia from parental exposure to radiation in the Sellafield work force and those displayed among the Japanese atomic bomb survivors. J Radiol Protection.

[b65-ehp0115-000138] Lord BI (1999). Transgenerational susceptibility to leukemia induction resulting from preconception, paternal irradiation. Int J Radiat Biol.

[b66-ehp0115-000138] Lowengart RA (1987). Childhood leukemia and parent’s occupational and home exposures. J Natl Cancer Inst.

[b67-ehp0115-000138] Lundell M, Holm LE (1996). Mortality from leukemia after irradiation in infancy for skin hemangioma. Radiat Res.

[b68-ehp0115-000138] Ma X, Buffler PA, Gunier RB, Dahl G, Smith MT, Reinier K (2002). Critical windows of exposure to household pesticides and risk of childhood leukemia. Environ Health Perspect.

[b69-ehp0115-000138] Mahoney MC, Moysich KB, McCarthy PL, McDonald RC, Stepanenko VF, Day RW (2004). The Chernobyl childhood leukemia study: background & lessons learned. Environ Health.

[b70-ehp0115-000138] Margolin JF Steuber CP Poplack DG 2001. Acute lymphocytic leukemia. In: Principles and Practice of Pediatric Oncology (Pizzo PA, Poplack DG, eds). Philadelphia:JB Lippincott, 489–544.

[b71-ehp0115-000138] McKinney PA, Alexander FE, Cartwright RA, Parker L (1991). Parental occupations of children with leukemia in west Cumbria, north Humberside, and Gateshead. BMJ.

[b72-ehp0115-000138] McLaughlin JR, King WD, Anderson TW, Clarke EA, Ashmore JP (1993). Paternal radiation exposure and leukaemia in offspring: the Ontario case-control study. BMJ.

[b73-ehp0115-000138] Meinert R, Kaletsch U, Kaatsch P, Schuz J, Michaelis J (1999). Associations between childhood cancer and ionizing radiation: Results of a population-based case-control study in Germany. Cancer Epidemiol Biomarkers Prev.

[b74-ehp0115-000138] Menegaux F, Baruchel A, Bertrand Y, Lescoeur B, Leverger G, Nelken B (2006). Household exposure to pesticides and risk of childhood acute leukaemia. Occup Environ Med.

[b75-ehp0115-000138] Michaelis J, Kaletsch U, Burkart W, Grosche B (1997). Infant leukaemia after the Chernobyl accident [Letter]. Nature.

[b76-ehp0115-000138] Miller RW (1967). Persons with exceptionally high risk of leukemia. Cancer Res.

[b77-ehp0115-000138] Miller RW (1968). Relationship between cancer and congenital defects: an epidemiologic evaluation. J Natl Cancer Inst.

[b78-ehp0115-000138] Moloney W (1955). Leukemia in survivors of atomic bombing. N Engl J Med.

[b79-ehp0115-000138] Moysich KB, Menezes RJ, Michalek AM (2002). Chernobyl-related ionising radiation exposure and cancer risk: an epidemiological review. Lancet Oncol.

[b80-ehp0115-000138] Murray R, Heckel P, Hempelmann L (1959). Leukemia in children exposed to ionizing radiation. N Engl J Med.

[b81-ehp0115-000138] Myers A, Cllyden A, Cartwright RA, Cartwright SC (1990). Childhood cancer and overhead powerlines: a case-control study. Br J Cancer.

[b82-ehp0115-000138] National Research Council

[b83-ehp0115-000138] Naumberg E, Bellocco R, Cnattingius S, Hall P, Boice JD, Ekbom A (2001). Intrauterine exposure to diagnostic x-rays and risk of childhood leukemia subtypes. Radiat Res.

[b84-ehp0115-000138] Neel JV, Schull WJ

[b85-ehp0115-000138] Noshchenko AG, Moysich KB, Bondar A (2001). Patterns of acute leukemia occurrence among children in the Chernobyl region. Int J Epidemiol.

[b86-ehp0115-000138] Parkin DM, Cardis E, Masuyer E, Friedl HP, Hansluwka H, Bobev D (1993). Childhood leukemia following the Chernobyl accident: the European Childhood Leukemia-Lymphoma Incidence Study (ECLIS). Eur J Cancer.

[b87-ehp0115-000138] Parkin DM, Clayton D, Black RJ, Masuyer E, Friedl HP, Ivanov E (1996). Childhood leukaemia in Europe after Chernobyl: 5 year follow-up. Br J Cancer.

[b88-ehp0115-000138] Petridou E, Trichopoulos D, Dessypris N, Flytzani V, Haidas S, Kalmanti M (1996). Infant leukemia after *in utero* exposure to radiation from Chernobyl. Nature.

[b89-ehp0115-000138] Pierce M (1936). Childhood leukemia. J Pediatr.

[b90-ehp0115-000138] Pinkel D (1993). Ethnicity and survival in children acute lymphoid leukemia. Leukemia.

[b91-ehp0115-000138] Pollock BH, DeBaun MR, Camitta BM, Shuster JJ, Ravindranath Y, Pullen DJ (2000). Racial differences in the survival of childhood B-precursor acute lymphoblastic leukemia: a Pediatric Oncology Group study. J Clin Oncol.

[b92-ehp0115-000138] Pui CH (1997). Acute lymphocytic leukemia. Pediatr Clin North Am.

[b93-ehp0115-000138] Pui CH (2000). Acute lymphocytic leukemia in children. Curr Opin Oncol.

[b94-ehp0115-000138] Pui CH, Sandlund JT, Pei D, Rivera GK, Howard SC, Ribeiro RC (2003). Results of therapy for acute lymphoblastic leukemia in black and white children. JAMA.

[b95-ehp0115-000138] Reynolds P, Behren JV, Gunier RB, Goldberg DE, Hertz A, Harnly ME (2002). Childhood cancer and agricultural pesticide use: an ecologic study in California. Environ Health Perspect.

[b96-ehp0115-000138] Reynolds P, Behren JV, Gunier RB, Goldberg DE, Hertz A, Smith DF (2003). Childhood cancer incidence rates and hazardous air pollutants in California: an exploratory analysis. Environ Health Perspect.

[b97-ehp0115-000138] Ries LAG, Kosary CL, Hankey BF, Miller BA, Clegg L, Mariotto A

[b98-ehp0115-000138] Rinsky RA (1981). Leukemia in benzene workers. Am J Ind Med.

[b99-ehp0115-000138] Rivard CE, Deadman JE (2003). Maternal occupational exposure to extremely low frequency magnetic fields during pregnancy and childhood leukemia. Epidemiology.

[b100-ehp0115-000138] Robison LL, Buckley JD, Daigle AE, Wells R, Benjamin D, Arthur DC (1989). Maternal drug use and risk of childhood non-lymphoblastic leukemia among offspring: an epidemiologic investigation implicating marijuana (a report from the Children’s Cancer Study Group). Cancer.

[b101-ehp0115-000138] Robison LL, Codd M, Gunderson P, Neglia JP, Smithson WA, King FL (1987). Birth weight as a risk factor for childhood acute lymphoblastic leukemia. Pediatr Hematol Oncol.

[b102-ehp0115-000138] Robison LL, Neglia JP (1987). Epidemiology of Down syndrome and childhood acute leukemia. Prog Clin Biol Res.

[b103-ehp0115-000138] Roman E, Watson A, Beral V, Buckle S, Bull D, Baker K (1993). Case-control study of leukemia and non-Hodgkin’s lymphoma among children aged 0–4 years living in west Berkshire and north Hampshire health districts. BMJ.

[b104-ehp0115-000138] Ron E (1998). Ionizing radiation and cancer risk: evidence from epidemiology. Radiat Res.

[b105-ehp0115-000138] Ron E, Modan B, Boice JD (1988). Mortality after radiotherapy for ringworm of the scalp. Am J Epidemiol.

[b106-ehp0115-000138] Ross JA, Davies SM, Potter JD, Robison LL (1994). Epidemiology of childhood leukemia with a focus on infants. Epidemiol Rev.

[b107-ehp0115-000138] Ross JA, Potter JD, Reaman GH, Pendergrass TW, Robison LL (1996). Maternal exposure to potential inhibitors of DNA topoisomerase II and infant leukemia (United States): a report from the Children’s Cancer Group. Cancer Causes Control.

[b108-ehp0115-000138] Ross JA, Potter JD, Shu XO, Reaman GH, Lampkin B, Robison LL (1997). Evaluating the relationship among maternal reproductive history, birth characteristics, and infant leukemia: a report from the Children’s Cancer Group. Ann Epidemiol.

[b109-ehp0115-000138] Ross JA, Severson RK, Swensen AR, Pollock BH, Gurney JG, Robison LL (1999). Seasonal variation in the diagnosis of childhood cancer in the United States. Br J Cancer.

[b110-ehp0115-000138] Rushton L, Romaniuk H (1997). A case-control study to investigate the risk of leukemia associated with exposure to benzene in petroleum marketing and distribution workers in the United Kingdom. Occup Environ Med.

[b111-ehp0115-000138] Sali D, Cardis E, Sztanyik L, Auvinen A, Bairakova A, Dontas N (1996). Cancer consequences of the Chernobyl accident in Europe outside the former USSR: a review. Int J Cancer.

[b112-ehp0115-000138] Sandler DP, Ross JA (1997). Epidemiology of acute leukemia in children and adults. Semin Oncol.

[b113-ehp0115-000138] Savitz DA, Chen J (1990). Parental occupation and childhood cancer: review of epidemiologic studies. Environ Health Perspect.

[b114-ehp0115-000138] Schnatter RA, Armstrong TW, Nicolich MJ, Thompson FS, Katz AM, Huebner WW (1996). Lymphohaematopoietic malignancies and quantitative estimates of exposure to benzene in Canadian petroleum distribution workers. Occup Environ Med.

[b115-ehp0115-000138] Schulz KF, Grimes DA (2002). Case-control studies: research in reverse. Lancet.

[b116-ehp0115-000138] Shearer P, Parham D, Kovnar E, Kun L, Rao B, Lobe T (1994). Neurofibromatosis type I and malignancy: review of 32 pediatric cases treated at a single institution. Med Pediatr Oncol.

[b117-ehp0115-000138] Shu XO, Linet MS, Steinbuch M, Wen WQ, Buckley JD, Neglia JP (1999a). Breast feeding and risk of acute childhood leukemia. J Natl Cancer Inst.

[b118-ehp0115-000138] Shu XO, Potter JD, Linet MS, Severson RK, Han D, Kersey JH (2002). Diagnostic x-rays and ultrasound exposure and risk of acute lymphoblastic leukemia by immunophenotype. Cancer Epidemiol Biomarkers Prev.

[b119-ehp0115-000138] Shu XO, Reaman GH, Lampkin B, Sather HN, Pendergrass TW, Robison LL (1994). Association of paternal diagnostic X-ray exposure with risk of infant leukemia. Cancer Epidemiol Biomarkers Prev.

[b120-ehp0115-000138] Shu XO, Ross JA, Pendergrass TW, Reaman GH, Lampkin B, Robison LL (1996). Parental alcohol consumption, cigarette smoking, and risk of infant leukemia: a Children’s Cancer Group study. J Natl Cancer Inst.

[b121-ehp0115-000138] Shu XO, Stewart P, Wen WQ, Han D, Potter JD, Buckley JD (1999b). Parental occupational exposure to hydrocarbons and risk of acute lymphocytic leukemia in offspring. Cancer Epidemiol Biomarkers Prev.

[b122-ehp0115-000138] Sorahan T, Lancashire R, Prior P, Stewart A (1995). Childhood cancer and parental use of alcohol and tobacco. Ann Epidemiol.

[b123-ehp0115-000138] Spector LG, Xie Y, Robison LL, Heerema NA, Hilden JM, Lange B (2005). Maternal diet and infant leukemia: the DNA topoisomerase II inhibitor hypothesis: a report from the children’s oncology group. Cancer Epidemiol Biomarkers Prev.

[b124-ehp0115-000138] Steiner M, Burkart W, Grosche B, Kaletsch U, Michaelis J (1998). Trends in infant leukemia in West Germany in relationship to in utero exposure due to Chernobyl accident. Radiat Environ Biophys.

[b125-ehp0115-000138] Stewart A, Webb K, Giles D (1956). Malignant disease in childhood and diagnostic irradiation i*n utero* [Letter]. Lancet.

[b126-ehp0115-000138] Swensen AR, Ross JA, Severson RK, Pollock BH, Robison LL (1997). The age peak in childhood acute lymphoblastic leukemia: exploring the potential relationship with socioeconomic status. Cancer.

[b127-ehp0115-000138] Swift M (1971). Fanconi’s anemia in the genetics of neoplasia. Nature.

[b128-ehp0115-000138] Toledano S, Lange B (1980). Ataxia-telangiectasia and acute lymphoblastic leukemia. Cancer.

[b129-ehp0115-000138] United Nations Scientific Committee on the Effects of Atomic Radiation

[b130-ehp0115-000138] Urquhart JD, Black RJ, Muirhead MJ, Sharp L, Maxwell M, Eden OB (1991). Case-control study of leukaemia and non-Hodgkin’s lymphoma in children in Caithness near the Dounreay nuclear installation. BMJ.

[b131-ehp0115-000138] Van Duijn CM, Van Steensel-Moll HA, Coebergh JW, Van Zanen GE (1994). Risk factors for childhood acute non-lymphocytic leukemia: an association with maternal alcohol consumption during pregnancy?. Cancer Epidemiol Biomarkers Prevent.

[b132-ehp0115-000138] Van Steensel-Moll HA, Valkenbueg HA, Van Zanen GE (1985). Childhood leukemia and parental occupation, a register-based case-control study. Am J Epidemiol.

[b133-ehp0115-000138] Vianna NJ, Kovasznay B, Polan A, Ju C (1984). Infant leukemia and paternal exposure to motor vehicle exhaust fumes. J Occup Med.

[b134-ehp0115-000138] Watson GM (1991). Leukaemia and paternal radiation exposure. Med J Aust.

[b135-ehp0115-000138] Westerbeek RM, Blair V, Eden OB, Kelsey AM, Stevens RF, Will AM (1998). Seasonal variations in the onset of childhood leukaemia and lymphoma. Br J Cancer.

[b136-ehp0115-000138] Westergaard T (1997). Birth characteristics, sibling patterns, and acute leukemia risk in childhood: a population-based cohort study. J Natl Cancer Inst.

[b137-ehp0115-000138] Wiemels JL, Leonard BC, Wang Y, Segal MR, Hunger SP, Smith MT (2002). Site-specific translocation and evidence of postnatal origin of the t(1;19) E2A-PBX1 fusion in childhood acute lymphoblastic leukemia. Proc Natl Acad Sci USA.

[b138-ehp0115-000138] Willis A, Lindahl T (1987). DNA ligase deficiency in Bloom’s syndrome. Nature.

[b139-ehp0115-000138] Woods WG, Roloff JS, Lukens JN, Krivit W (1981). The occurrence of leukemia in patients with the Shwachman syndrome. J Pediatr.

[b140-ehp0115-000138] Yeazel MW, Buckley JD, Woods WG, Ruccione K, Robison LL (1995). History of maternal fetal loss and increased risk of childhood acute leukemia at an early age, a report from the Childrens Cancer Group. Cancer.

[b141-ehp0115-000138] Yoshimoto Y, Kato H, Schull WJ (1988). Risk of cancer among children exposed *in utero* to A-bomb radiations, 1950–84. Lancet.

[b142-ehp0115-000138] Yoshimoto Y, Neel JV, Schull WJ (1990). Malignant tumors during the first 2 decades of life in the offspring of atomic bomb survivors. Am J Hum Genet.

[b143-ehp0115-000138] Zahm SH, Devesa SS (1995). Childhood cancer: overview of incidence trends and environmental carcinogens. Environ Health Perspect.

[b144-ehp0115-000138] Zahm SH, Ward MH (1998). Pesticides and childhood cancer. Environ Health Perspect.

[b145-ehp0115-000138] Zipf TF Berg S Roberts WM Poplack DG Steuber CP Bleyer WA 2000. Childhood leukemias. In: Clinical Oncology (Abeloff MD, Armitage JO, Lichter AS, Niederhuber JE, eds). 2nd ed. Philadelphia:Churchill Livingston, 2402–2429.

