# Errata

**Published:** 2010-09

In the conclusion of the article “Risk Factors for Acute Leukemia in Children: A Review” by Belson et al. [ Environ Health Perspect 115:138–145 (2007)], benzene was incorrectly noted to be associated with the development of childhood acute lymphocytic leukemia (ALL) and acute myelogenous leukemia (AML). The authors note that although benzene is a known carcinogen associated with adult leukemia, in general, it is not associated with the development of childhood AML or ALL. Ionizing radiation is the only environmental exposure strongly associated with the development of childhood leukemia.

